# Follicular proliferation TIR3B: the role of total thyroidectomy vs lobectomy

**DOI:** 10.1186/s12893-019-0485-9

**Published:** 2019-04-24

**Authors:** Andrea Polistena, Alessandro Sanguinetti, Roberta Lucchini, Stefano Avenia, Sergio Galasse, Raffaele Farabi, Massimo Monacelli, Nicola Avenia

**Affiliations:** 1 General Surgery and Surgical Specialties Unit, S. Maria University Hospital Terni and University of Perugia, Medical School, Terni, Italy; 2 Pathology Unit, S. Maria University Hospital Terni and University of Perugia, Medical School, Terni, Italy

**Keywords:** Follicular proliferation, TIR3B, Thyroid cancer, Malignancy, Surgery, Total thyroidectomy, Lobectomy

## Abstract

**Background:**

TIR3B thyroid nodules are considered to be at risk of malignancy (15–30%) but guidelines recommend conservative surgery with lobectomy with primary diagnostic porpoise. Risk stratification mainly based on ultrasound, elastography and genetic mutations usually may influences the surgical approach.

**Methods:**

We retrospectively analyzed 52 cases of TIR3B underwent between 2015 and 2017 total thyroidectomy (TT) and lobectomy (L), focusing mainly on the observed rate of malignancy. Chi-squared test and Fisher’s exact probability test were used for analysis, considering a *P* values less than 0.05 as significant.

**Results:**

Out of 52 patients 49 underwent TT and 3 L. In TT group a multinodular goiter was associated in 67.3% of patients. Malignancy rate was 81.6 and 33.3% respectively after TT and L (P 0.003). Multicentric and contralateral tumors were detected respectively in 36.7% and in 32.6% of patients underwent TT. No main post-operative complications were registered.

**Conclusions:**

Ultrasound and elastography are useful to define within the TIR3B group those lesions at higher risk and therefore requiring a more radical approach. TT seems an appropriate approach to TIR3B lesions, especially in multinodular goiter, considering the incidence of malignancy with probably higher rate than previously reported.

## Background

In 2014, the Italian Society for Anatomic Pathology and Cytology (SIAPEC) together with the Italian Thyroid Association (AIT) modified the previous thyroid cytology classification of 2007, by replacing the TIR3 class with two new subclasses, TIR3A and TIR3B comparable respectively to the Bethesda category AUS/FLUS and FN/SFN [1–4]. TIR3B group includes those cases with mild/focal nuclear atypia suggestive of papillary carcinoma and therefore considered to be at higher risk of malignancy compared to TIR3A [1, 2]. The incidence of the TIR3B is expected to be lower than 10% with an associated malignancy rate of 20–30% [5]. Recent publications show instead an increased rate of TIR3B lesions with associated higher malignancy than expected up to 50–60% [6]. This consideration might suggest an approach to TIR3B nodules different to what stated by international guidelines, included those by the American Thyroid Association (ATA), which recommend conservative surgery with lobectomy with primary diagnostic porpoise [7]. Risk stratification based on ultrasound (US) pattern, real-time elastography (RTE), size, clinical behaviour and genetic mutations helps, in the clinical practice, in the choice of the more appropriate surgery [7]. The above considerations must be analyzed in the general scenario of the recent approach to thyroid cancer for which after a risk stratification less invasive forms and microcarcinoma are referred to observation or lobectomy rather than total thyroidectomy as in the past [8]. We report our institutional experience (2015–2017) in the management of TIR3B thyroid nodules focusing on the role of total thyroidectomy for high risk follicular proliferative lesions.

## Methods

We retrospectively analyzed 52 cases of follicular proliferation classified as TIR3B according to SIAPEC 2014, treated between January 2015 and December 2017 in the Unit of Endocrine Surgery, *S. Maria* University Hospital, Terni, University of Perugia, Italy, referral centre of Umbria region (middle Italy), by the same surgical team, with standard surgery for both total thyroidectomy (TT) and lobectomy (L). The diagnostic work out adopted standard technique as US, RTE and cytology. Molecular analysis was available in a limited number of patients (5 cases) and therefore not considered for the present study. Risk stratification was done in all cases. US features of the nodule were evaluated in terms of cancer risk according to ATA guidelines [9]. Size larger than 2 cm, was either considered a risk factor. High elasticity index as previously described [10, 11] was used as a further criteria of suspicious malignancy. Main indications for TT was evidence of high risk TIR3B lesion in a multinodular goiter, furthermore patient’s preference for a radical treatment and refusal of further eventual contralateral surgery were considered even in unilateral goiter. Main indication to thyroid L was the evidence of uninodular low risk TIR3B goiter with adequate informed consent about the possibility for eventual further surgery and in cases with patient refusing to undergo total thyroidectomy for an indeterminate lesion. To all patients was provided a specific informed consent focusing on specific risk of cancer in the TIR3B lesion and the possibility of reoperation in case of malignancy at final histology. All histological examinations after TT or L were referred to expert dedicated pathologist.

The statistical comparison between groups was performed by using chi-squared test and Fisher’s exact probability test. *P* values less than 0.05 were considered as statistically significant.

## Results

Out of 52 patients, 34 females and 28 males, presenting at admission a TIR3B lesion, 49 underwent TT and only 3 thyroid L. In TT group a multinodular bilateral goiter was associated in 33 (67.3%) patients, all patients of the L group presented uninodular goiter. Malignancy was detected in 81.6% (*n* = 40) and in 33.3% (*n* = 1) respectively after TT and L (P 0.003), with a cumulative malignancy rate of 78.8% (41 out of 52 patients). Only 6 patients out of 49 undergone TT, presented a an isolated low risk, T2 (< 4 cm) carcinoma. The patient with carcinoma after L was treated with contralateral surgery after 2 months. Main size of the TIR3B nodules was 1.7 cm. Size larger than 2 cm was not significantly associated to presence of carcinoma (P 0.057) in the TT group. Among malignant TIR3B a microcarcinoma, less than 1 cm in main diameter, was detected in 15 (36.5%) patients. Out of 49 patients, TT allowed detection of multicentric tumor in 18 (36.7%) cases and in 16 (32.6%) a contralateral carcinoma was detected. The principal histology detected was follicular variant of papillary carcinoma in 37 cases out of 41 (92.6%). High preoperative risk of the TIR3B lesions stratified on US feature, RTE and clinical behaviour fitted the diagnosis of carcinoma in 36/41 patients (87.8%). No main post-operative complications were registered. No recurrent laryngeal nerve lesions were observed after both TT and L. Only 3 cases of transient hypoparathyroidism were observed in the TT group.

## Discussion

The new SIAPEC classification of 2014, assign to TIR3B lesions, referable to the Bethesda FN/SFN group, an higher risk of malignancy compared to TIR3A, based on the evidence of mild/focal nuclear atypia [1, 2]. Features of capsule invasion and microvascular emboli move the histological diagnosis to a follicular or a follicular variant of papillary carcinoma, excluding the benign follicular adenoma or other benign conditions. International guidelines by ATA recommend for TIR3B lesions conservative surgery with lobectomy with primary diagnostic porpoise [7], based on a literature reported malignancy rate ranging between 20 and 30% [12]. In our series patients presenting TIR 3B nodules were mainly approached by TT. The appropriateness of this surgical strategy is supported by the increased rate, up to 80%, of malignant lesions found in our analysis at final histology, compared to the expected above rate reported in the literature [12]. This evidence in similar to what reported in recent publications showing an increased rate of TIR3B lesions with associated higher malignancy than expected up to 50–60% [6]. Similarly our series includes a 67.3% of concomitant goiter in the examined patients and an elevated incidence of multifocal and bilateral tumors (respectively 36.7 and 32.6%). There are several potential explanations of this evidence. First of all we must consider the specific features of the examined population and of the study setting. Umbria region, middle Italy and specifically the district of Terni (228.218 inhabitants) presents with one of the highest rate of endemic goiter and with a significant higher rate of malignant tumors including thyroid differentiated carcinoma, compared to other areas of the country, according to the data of the local tumor registry [13]. This might affect, as a selection bias, the high incidence of cancer in TIR3B lesions observed in our patients. Furthermore the present study was conducted in a referral centre for endocrine disease, with specific attitude for the treatment of thyroid cancer and somehow it might represent a bias for the malignancy rate, due to a casual patients selection in the examined population. Moreover a multidisciplinary management determines an high standard in preoperative patients selection by dedicated endocrinologist and pathologist. As a matter of fact the latter probably developed a specific attitude in the cytological and histological examinations of the thyroid fine needle aspiration samples and in the final pathology on the excised specimen. This might imply a more appropriate inclusion in the TIR3B class only of those nodules presenting a real atypia in the follicular proliferation, with consequent selection of those patients with an high suspicion of cancer and consequently exclusion of those with low risk features. Another important point must be referred to the final histological analysis. Quality of the pathological final report must be attested by the number of sections performed, which must be at least one every 5 mm, in order to achieve, especially in large lesions an appropriate number of slides [1, 11, 12, 14]. Fundamental requirement for the diagnosis of thyroid cancer is the presence of capsule invasion, whose detection needs an higher number of section the larger the nodules are, in order to examine all capsule surface. Some missed final diagnosis can occur if this technical approach is not respected. Pathological examination might therefore reflect another bias with increased rate of malignant lesions compared to what usually reported in the literature. As a consequence of these data in our opinion there are several points to be raised in the definition of the optimal treatment for TIR3B nodules. First of all the presence of a concomitant multinodular bilateral goiter could represent the main indication for a TT, compared to isolated TIR3B single nodules, which although rarer, might represent instead an ideal indication for L, after an adequate and mandatory risk stratification. Molecular study is not always available in our setting and this limits the risk analysis usually referred only to US, RTE [2, 11] and clinical features thus limiting the specificity of the preoperative workout [7, 15, 16]. Nevertheless we observed an high correspondence of high risk TIR3B nodules for their US and RTE pattern with the evidence of cancer at final histology. The high incidence of multifocal and bilateral tumor observed in this series, in our opinion might suggest to refer patients with high risk TIR3B lesions to a TT, as we would do for the treatment of a suspicious or evident thyroid cancer. In this scenario of thyroid lesions at risk for cancer, we must consider what is the actual trend in the treatment of thyroid cancer, mostly followed in the United States according to the most recent version of ATA guidelines, indicating that L is adequate treatment for low risk, T2 (< 4 cm), clinically N0 patients [8, 17–19]. In our experience only 6 patients out of 49 presented a an isolated low risk carcinoma nevertheless they all underwent TT, according to clinical evaluation of suspicious lesion and following patient’s preference. In such cases actually a less radical approach would be sufficient considering the above new trend in surgical management of low risk subgroup of thyroid cancer. Nevertheless large studies comparing the two different approaches, in terms of prognosis, are still missing so far. The arguments for choosing radical surgery instead of a limited procedure for a papillary thyroid carcinoma were very well reviewed by Miccoli and Bakkar [16]. In the management of thyroid cancer, points in favour of TT are: treatment of multicentric and bilateral tumors (as an hallmark of papillary carcinoma), improved disease-free survival, less local recurrence which are associated to reoperative complications and decreased survival, improved detection and elimination by radioiodine treatment of persistent and metastatic disease, improved surveillance by thyroglobulin dosage, contained primary surgical complications in high volume centres not differing from those observed after L and of course elimination of the risk of completion surgery. As in our experience the high incidence of contralateral tumors associated to a follicular variant of papillary carcinoma, similarly described by Sullivan et al. in the 35% of their cases [20], supports the rationale of performing radical surgery in order to reduce the misdiagnosis and either the need for further surgeries. As opposite arguments, L might be preferred since occult residual cancer foci are usual non-progressive and without implication on morbidity and survival [16]. The same authors [16] supporting conservative treatment, consider that in case of recurrence completion surgery after L addict no further morbidity compared to primary radical surgery, that L reduces of potential risk of primary TT according to the centre experience and that there are limited indications to radioiodine only in selected cases with no need for radical primary surgery as first step. Nevertheless other authors point on the potential consequences following thyroid L also for indeterminate lesions, in terms of risk of hypoparathyroidism (up to 20% of cases) with consequent need for calcium/vitamin D replacement (36.7% of cases) [21] and even the risk of hypothyroidism and the consequent need of substitutive/suppressive therapy (32.6% of cases) [22] with the related increased costs.

The decision on what is the best treatment to propose to our TIR3B patients, although affected by several variables and sometimes weak risk stratification criteria, represents both for patient and surgeon a decisional conflict [23]. This conflict is evident in clinical practice and can be measured by specific analysis such as the Decisional Conflict Scale used by Taylor and al. in the setting of the indeterminate thyroid lesion at fine needle aspiration [23]. Shared decision-making is crucial in this clinical situation. Collaboration between healthcare providers and their patients is required to understand the treatment options and have knowledge of their risks and benefits. At the same time, shared decisions should consider the patients’ own preferences and values in decision-making [24]. The sum of the above criteria mainly including molecular analysis, in an improving future perspective, might help in personalize surgery for TIR3B lesions [25]. Our institutional large experience in the management of thyroid cancer supports total thyroidectomy in preoperatively diagnosed patients based also on limited complication rates. This approach probably affected the tendence to propose TT as a standard of care also for TIR3B lesions in presence of high risk clinical and US criteria and always if a multinodular goiter is present. Our data might be affected by the limited number of examined cases but, also based on previous experience [6], TIR3B is anyway a lesion with a significant high risk for cancer. When cancer is present, although not associated to severe prognosis since it usually shows a general benign course especially for small nodules, it remains a cancer with potential aggressiveness [26]. Emphasis must be placed on the appropriateness of care and avoidance of over- and under-treatment and mainly on avoidance of reoperation for completion thyroidectomy which is usually required, as in our series, when a TIR3B nodule is revealed as malignant at histopatology according to the international guidelines [27].

## Conclusions

Our data and recent publications show an increased rate of TIR3B lesions with associated higher malignancy rate than expected. This evidence might recommend a radical approach to TIR3B nodules by TT differently to what stated by ATA guidelines which considers conservative surgery with L with diagnostic primary porpoise. Risk stratification based on US pattern, size, clinical behaviour, genetic mutations supports, in clinical practice, the choice of the more appropriate surgery. The presence of concomitant multinodular goiter and risk for bilateral and multicentric tumors are the main factors supporting the choice for a TT in TIR3B. The above considerations must be otherwise analyzed in the general scenario of the recent approach to thyroid cancer for which after a risk stratification, less invasive forms and microcarcinoma are referred to observation or L rather than to TT as in the past.

## Abbreviations

AIT: Italian Thyroid Association
ATA: American Thyroid Association
L: Lobectomy
RTE: Real-time elastography
SIAPEC: Society for Anatomic Pathology and Cytology
TT: Total thyroidectomy
US: Ultrasound
